# Chromosome-Level Genome Assembly of the Asian Red-Tail Catfish (*Hemibagrus wyckioides*)

**DOI:** 10.3389/fgene.2021.747684

**Published:** 2021-10-12

**Authors:** Feng Shao, Huamei Pan, Ping Li, Luyun Ni, Yuan Xu, Zuogang Peng

**Affiliations:** ^1^ Key Laboratory of Freshwater Fish Reproduction and Development (Ministry of Education), Southwest University School of Life Sciences, Chongqing, China; ^2^ College of Fisheries, Southwest University, Chongqing, China

**Keywords:** *Hemibagrus wyckioides*, genome, HiFi reads, comparative genomics, asssembly

## Introduction

Aquaculture plays a vital role in food security and economic stability worldwide (Houston et al., 2020). Selective breeding to genetically improve production traits has great potential in increasing the efficiency of aquaculture and reducing its environmental footprint (such as habitat destruction and infectious disease outbreaks). To achieve this, we require fish species with excellent economic traits and high-quality genomic data that can be applied at all stages of domestication for ongoing genetic improvement (Houston, et al., 2020).

Siluriformes (catfish) is an order of major aquaculture species worldwide, especially in China, the United States, and Vietnam (De Silva and Phuong, 2011; Zhong et al., 2016; Kumar et al., 2020). Red-tail catfish (*Hemibagrus wyckioides*), belonging to the family Bagridae, initially possess a white caudal fin that becomes bright red when it reaches approximately 15 cm. The red-tail catfish is the largest Bagridae fish in body size and weight, reaching 130 cm and 80 kg, respectively (Ng, 1999). Red-tail catfish were originally distributed throughout the Mekong River. In China, they are now only distributed in Yunnan Province. Here, it is a famous indigenous fish due to a variety of excellent economic traits such as high protein content, strong disease resistance, easy domestication, and better production performance; it also has ornamental value (Zhou et al., 2021). Therefore, it has recently become an important aquaculture species in China (Zhou et al., 2019; Zhou et al., 2021). *H. wyckioides* exhibits marked sex dimorphism in growth, with the males growing much faster than females. Sexual maturation takes over 3 years and a lack of selective breeding has resulted in a decline in the growth rate of red-tail catfish (Zhou et al., 2021). Thus, it is essential to construct a reference genome of *H. wyckioides* and establish a breeding program to improve the economic characteristics, maintaining and developing the industry, to ultimately create an economically viable local fishing industry.

## Data

In total, 41 Gb of clean reads (Illumina reads after quality control and trimming, the sequencing data used in the study detailed in Supplementary Table S1) were used to analyze the genome size and heterozygosity in *H. wyckioides* using *k-mer* analysis. Based on 26, 507, 871, 386 17-mers and a peak 17-mer depth of 34, the estimated heterozygosity rate was ∼0.3%, and the estimated genome size of *H. wyckioides* was ∼779 Mb (Supplementary Figure S1). It is the largest published genome size of Bagridae fish, as compared to that of the other two species; that of *Pseudobagrus fulvidraco* is ∼718 Mb (Gong et al., 2018) and that of *Leiocassis longirostris* is ∼689 Mb (He et al., 2021).

**FIGURE 1:**
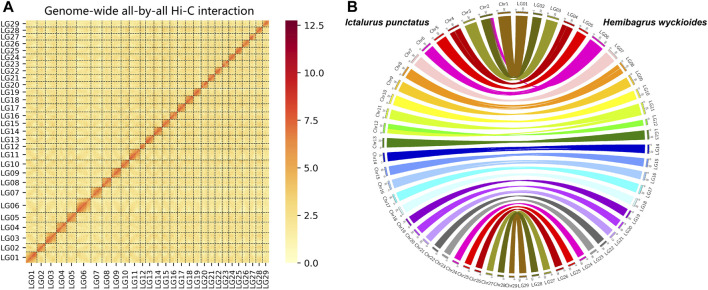
Pseudo-chromosome construct and comparative genomic analysis. **(A)** *Hemibagrus wyckioides* genome contig contact matrix using Hi-C data. **(B)** Genomic synteny of *H. wyckioides* and *Ictalurus punctatus*.

We produced 74.9 Gb of ONT (Oxford Nanopore Technologies) long reads and 6.7 Gb of PacBio HiFi reads. We used these data to construct our initial assembly. We obtained a 789.8 Mb genomic DNA sequence *via* assembly with a contig N50 length of 22.1 Mb (Supplementary Table S2). The long read assembly results consisted of 176 contigs, and the longest contig was 37.9 Mb (Supplementary Table S2). BUSCO (Simao et al., 2015) was used to assess the completeness of the assembled genome. Approximately 95.9% of the complete genes were detected in the genome of *H. wyckioides* (Supplementary Table S3). In addition, the average proportion of RNA-seq short reads were mapped to the assembled genome from different tissues is over 90% (Supplementary Table S4). Finally, we used the Hi-C technique to anchor the assembly contigs at the 29 chromosome level for *H. wyckioides*. We found that 136 contigs were successfully anchored in 29 chromosomes (Figure 1A). This result is consistent with the chromosome number records obtained by cytogenetic analysis (Supiwong et al., 2014), representing 97.7% of all scaffold nucleotide bases. The total assembly size of the chromosomes was ∼771.6 Mb (Supplementary Table S5).

We aligned the entire genomic DNA sequences from *I. punctatus* and *H. wyckioides* to create the same chromosome numbering system for both species. More importantly, this greatly improves the usability of the data in future comparative genomics analyses, and the results showed that these species possess good collinearity (Figure 1B), which further demonstrates the reliability of the genomic data produced in this study. In total, 316, 847, 809 bp repeat sequences (40.12%) were identified. Overall, the combined homology-based and *de novo* prediction results indicated that TEs accounted for 34.59% of the assembled genome (Supplementary Table S6). Additionally, long terminal repeats, long interspersed nuclear elements, short interspersed nuclear elements, and DNA transposons occupied 4.84, 1.17, 6.13, and 19.09% of the assembled genome, respectively. Similar to most fish genomes, DNA transposons constituted a large proportion (Shao et al., 2019). *H. wyckioides* has the highest content of transposons among the published fish of the family Bagridae (Gong et al., 2018; He et al., 2021), which is consistent with the view that the larger the fish genome, the higher the content of transposons (Shao et al., 2019).

For genome annotation, 22,794 protein-coding genes were predicted in the *H. wyckioides* genome. Compared with other previously published catfish annotated information, the statistical results of the distribution showed that the CDS length, the number of exons, the length of exons, and the length of introns in the genes of *H. wyckioides* were consistent with the distribution trends of related species such as *G. maculatum*, *P. fulvidraco*, and *B. yarrelli* (Supplementary Figure S2). The BUSCO gene prediction of the existing genome sequence utilized the Actinopterygii_odb10 single-copy homologous gene. Approximately 95% of complete gene components were found in this gene set. This result indicates that most of the conserved genes were well predicted and the prediction results were relatively reliable (Supplementary Table S7). Finally, 21,142 genes were annotated in ≥1 of the databases (KOG, KEGG, NR, SwissProt, GO), and up to 92.75% of the genes were functionally annotated (Supplementary Table S8).

**FIGURE 2:**
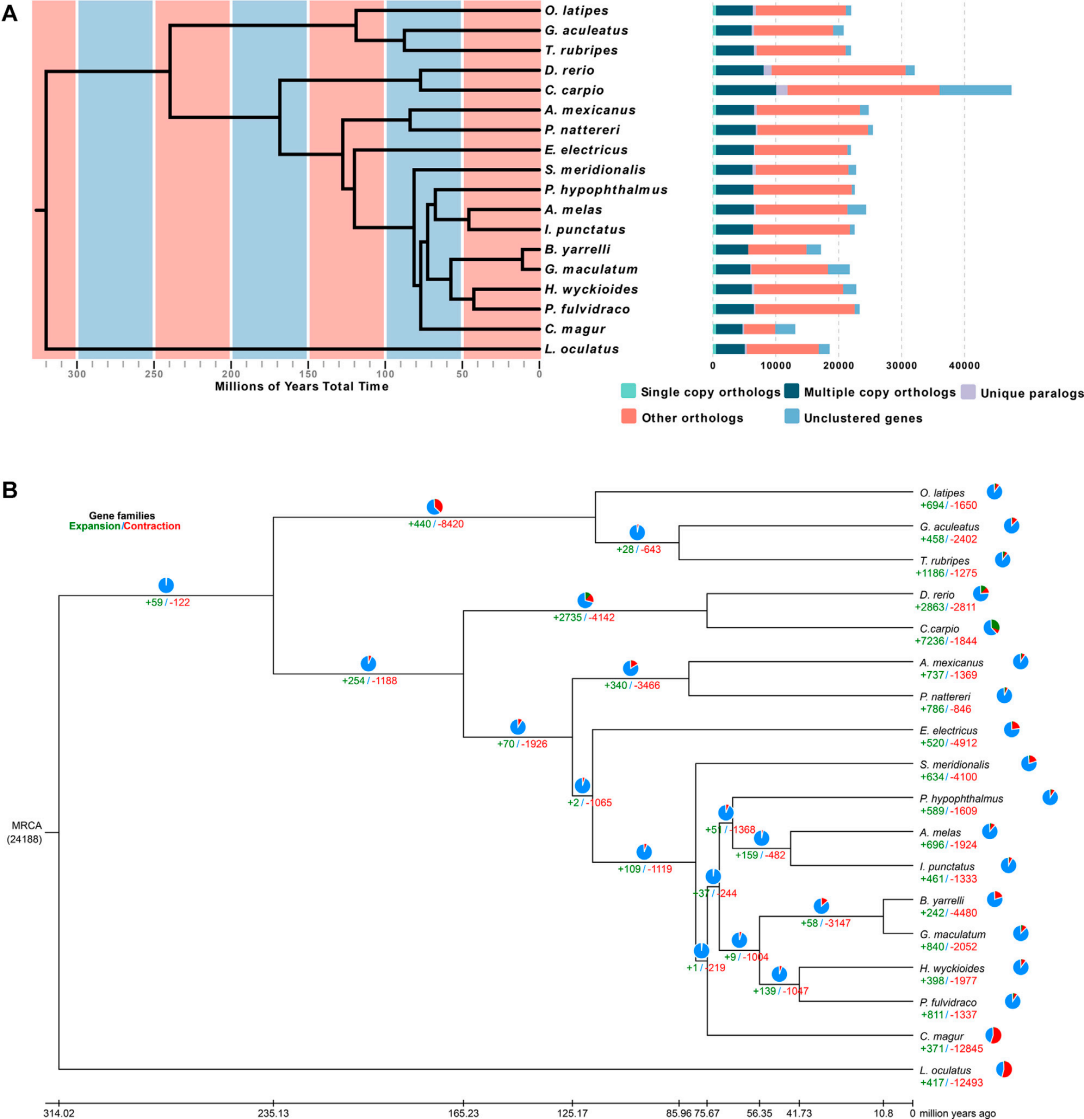
Phylogenetic and evolutionary analysis of *Hemibagrus wyckioides*. **(A)** Divergence time estimates and gene clusters in *H. wyckioides* and other species. **(B)** Expansion and contraction of *H. wyckioides* gene families. MRCA: most recent common ancestor; pie charts and numbers below represent the proportion and specific values of the gene families of expansion (green) and contraction (red), respectively.

To determine the evolutionary relationships among *H. wyckioides* and other vertebrates, a phylogenetic tree was constructed using the 507 single-copy orthologous genes from 17 other vertebrate genomes (Figure 2A). *L. oculatus* was used as the outgroup. *H. wyckioides* and *P. fulvidraco* (which belong to the Bagridae family) formed a branch. Nine species of Siluriformes formed a monophyletic group. Siluriformes and Gymnotiformes formed sister groups. Otophysa fish were grouped together. Asian species (*H. wyckioides*, *P. fulvidraco*, *G. maculatum*, and *B. yarrelli*) clustered into one group and North American species (*I. punctatus* and *A. melas*) clustered into sister groups. This result is consistent with the “Big Asia” branch views suggested by Sullivan (Sullivan et al., 2006). We then created a time tree, and the estimated divergence time between *H. wyckioides* and *P. fulvidraco* was ∼41.73 Mya (Figure 2B; Supplementary Figure S3). In addition, the divergence time between Siluriformes and Gymnotiformes was ∼117.74 Mya. The divergence time between Anotophysi and Otophysa was ∼235.12 Mya.

We identified 398 expansion gene families and 1,977 contraction gene families in *H. wyckioides* (Figure 2B). Expansion gene families were enriched in 22 GO (Supplementary Table S9) categories and 33 KEGG pathways (Supplementary Table S10), most of which were related to nutrient metabolism (carbohydrates, proteins, and fats). This result provides insight for future studies on *H. wyckioides* growth and nutrient metabolism. Contraction gene families were enriched in 19 GO (Supplementary Table S11) categories and nine KEGG pathways (Supplementary Table S12), most of which were related to ion transport, cell interaction, and proteolysis.

## Materials and Methods

### Sample Collection, Library Construction, and Sequencing

Samples for genome sequencing of female *H. wyckioides* were collected from the Lancang River System, Xishuangbanna Prefecture, Yunnan Province, China (21°29′30.74″ N, 101°34′14.28″ E). The fin, blood, brain, gills, heart, head kidney, liver, muscle, and spleen were collected and immediately frozen in liquid nitrogen. Blood samples were collected and DNA was prepared using the QIAGEN^®^ Genomic kit (Cat NO./ID: 13343, QIAGEN). The qualified libraries (200–400 bp) were sequenced using MGISEQ 2000. Raw reads were first filtered using a fastp (Chen et al., 2018) preprocessor (set to default parameters). For the ONT library preparations, the long DNA fragments were selected using the BluePippin system (Sage Science, United States). Sequencing was performed using a Nanopore PromethION sequencer (Oxford Nanopore Technologies, United Kingdom). The files were initially converted from FAST5 into FASTQ format using Guppy (Wick et al., 2019). The raw reads in fastq format with a mean_qscore_template <7 were then filtered. SMRTbell target size libraries (10–20 kb) were constructed for sequencing according to PacBio’s standard protocol (Pacific Biosciences, CA, United States). Sequencing was performed using a PacBio Sequel II. Raw data were analyzed using Smrtlink software (https://www.pacb.com/support/software-downloads/).

The RNA analyses involved nine tissues (fin, brain, gills, heart, head kidney, liver, muscle, and spleen) that were extracted using an RNeasy Plus Mini Kit (Qiagen). The Illumina paired-end sequencing (validated RNA samples, Illumina HiSeq4000, 150 bp) involved preparing a complementary DNA (cDNA) library using a TruSeq Sample Preparation Kit (Illumina). The qualified RNA from the nine tissues was mixed in equal amounts and reverse-transcribed using SQK-PCS109 (Oxford Nanopore Technologies) for the ONT library preparations and sequencing (Nanopore PromethION). The raw data were filtered in the same manner as DNA.

### Genomic Features From K-Mer Analysis and Long Read Assembly

Quality-filtered reads were subjected to 17-*mer* frequency distribution analysis using the Jellyfish program (Marcais and Kingsford, 2011). We analyzed the 17-*mer* depth distribution from clean sequencing reads in FindGSE software (Sun et al., 2018). The simulation data results of *Arabidopsis thaliana* were further combined with different heterozygosity levels and the frequency peak distribution of the 17 *k-mer* using pIRS (Hu et al., 2012) to estimate the heterozygosity and the repeat content within the *H. wyckioides* genome. The long read assembly was constructed using NextDenovo (reads_cutoff:1k, seed_cutoff:32k, https://github.com/Nextomics/NextDenovo). To improve the accuracy of the assembly, the contigs were refined with default parameters in Racon for the long reads and Nextpolish using Illumina for the short reads.

### Chromosomal-Level Genome Assembly by Hi-C and Assessment

To anchor the hybrid scaffolds onto the chromosome, genomic DNA was extracted from the blood of *H. wyckioides* for the Hi-C library, and sequencing (Illumina MGI-2000) was performed. The scaffolds were further clustered, ordered, and oriented onto chromosomes with LACHESIS (Korbel and Lee, 2013), with parameters CLUSTER_MIN_RE_SITES = 100, CLUSTER_MAX_LINK_DENSITY = 2.5, CLUSTER NONINFORMATIVE RATIO = 1.4, ORDER MIN N RES IN TRUNK = 60, and ORDER MIN N RES IN SHREDS = 60. Finally, the placement and orientation errors exhibited by obvious discrete chromatin interaction patterns were manually adjusted.

BUSCO and RNA-seq data mapping were used to evaluate our genome assembly. BUSCO was used to assess the completeness of the genome assembly by searching for the single-copy genes conserved across Actinopterygii in the *H. wyckioides* genome. We used hisat2 (Kim et al., 2015) to map RNA-seq short reads to the *H. wyckioides* genome. Furthermore, to compare the chromosome-level genome of *H. wyckioides* in this study with a reported chromosome-level genome of *I. punctatus* (Liu et al., 2016), we also performed a synteny analysis of these two genome assemblies using MUMmer (Marcais et al., 2018), only considering the reliable aligned regions more than 1 Mb in length. Circos plot distributions of homologous sequence pairs were plotted using Circos (Krzywinski et al., 2009).

### Annotation of Repetitive Elements

We first annotated the tandem repeats using the software GMATA (Wang and Wang 2016) and TRF (Benson, 1999). For transposable element (TE) annotation, an *ab inito* repeat library for *H. wyckioides* was initially predicted using MITE-hunter (Han and Wessler, 2010) and RepeatModeler (Flynn et al., 2020) with default parameters, and LTR_FINDER (Xu and Wang, 2007), LTRharverst (Ellinghaus et al., 2008), and LTR_retriver (Ellinghaus et al., 2008) were also included in the *H. wyckioides* genome. The obtained library was then aligned to repbase (Bao et al., 2015) using TEclass (Abrusan et al., 2009) to classify the type of each repeat family. RepeatMasker (https://www.repeatmasker.org/) was applied to search for known and novel TEs by mapping sequences against the *de novo* repeat library and repbase TE library.

### Gene Prediction and Functional Annotation

GeMoMa (Keilwagen et al., 2016) was used to align the homologous peptides from related species (*Danio rerio*, *Oryzias latipes*, *Takifugu rubripes*, *Homo sapiens*, *P. fulvidraco*, *Glyptosternon maculatum*, *Bagarius yarrelli*, and *Ictalurus punctatus*) to the assembly to obtain the gene structure information using homolog prediction. The RNA-seq-based gene prediction involved filtered RNA-seq short reads being aligned to the reference genome using STAR (Dobin et al., 2013) with default parameters. The transcripts were assembled using StringTie (Pertea et al., 2015). Full-length reads were identified and oriented from sequencing reads using the Pychopper tool (https://github.com/nanoporetech/pychopper) with default parameters. Full-length reads were aligned to the *H. wyckioides* reference genome using minimap2 (Li, 2018) with “-ax splice -uf”. The aligned full-length reads were clustered using pinfish software (https://github.com/nanoporetech/pinfish) following Nanopore’s Official recommendation. Redundancy was removed using cDNA_Cupcake software (https://github.com/Magdoll/cDNA_Cupcake) and polished using a reference genome sequence. The assembled transcripts based on full-length and short reads were merged with the open reading frames (ORFs) and were predicted using PASA (Haas et al., 2008). The *de novo* prediction involved RNA-seq reads being assembled for *de novo* using StringTie (Pertea et al., 2015) and analyzed with PASA (Haas et al., 2008) to produce a training set. AUGUSTUS (Stanke et al., 2008) with default parameters was used for the *ab initio* gene prediction with the training set. Finally, EVidenceModeler (Haas et al., 2008) was used to produce an integrated gene set in which genes with TEs were removed using the TransposonPSI package (http://transposonpsi.sourceforge.net/) and the miscoded genes were further filtered. BUSCO was used to assess the accuracy of gene prediction by searching for the single-copy genes conserved across Actinopterygii among the predicted genes in the assembly.

Gene function information, motifs, and domains of their proteins were assigned and compared with public databases, including SwissProt (UniProt Consortium, 2021), NR (https://www.ncbi.nlm.nih.gov/refseq/about/nonredundantproteins/), KEGG (Kanehisa et al., 2014), KOG (Tatusov et al., 2003), and GO (The Gene Ontology Consortium, 2017). The putative domains and GO terms of the genes were identified using the InterProScan (Jones et al., 2014) program with default parameters. For the other four databases, BLASTP (Camacho et al., 2009) was used to compare the EvidenceModeler-integrated protein sequences against the four well-known public protein databases with an E-value cutoff of 1e-5.

### Evolutionary and Comparative Genomic Analyses

We identified homologous relationships among *H. wyckioides* and other species (*I. punctatus*, *G. maculatum*, *Pangasianodon hypophthalmus*, *P. fulvidraco*, *Clarias magur*, *B. yarrelli*, *Ameiurus melas*, *Silurus meridionalis*, *Electrophorus electricus*, *Astyanax mexicanus*, *Pygocentrus nattereri*, *Cyprinus carpio*, *D. rerio*, *O. latipes*, *T. rubripes*, *Lepisosteus oculatus*, and *Gasterosteus aculeatus*) by downloading their protein sequences and aligned them using OrthoMCL (Li et al., 2003).

Based on the orthologous gene sets identified with OrthoMCL (Li et al., 2003), molecular phylogenetic analysis was performed using the shared single-copy genes. Each ortholog group was multiple aligned using MAFFT (Yamada et al., 2016). Poorly aligned sequences were then eliminated using Gblocks (http://molevol.cmima.csic.es/castresana/Gblocks.html), and the GTRGAMMA substitution model in RAxML (Stamatakis, 2014) were used for phylogenetic tree construction with 1,000 bootstrap replicates. Three fossil calibration times were obtained from the TimeTree database (http://www.timetree.org/), the control time with the divergence times are provided in Supplementary Table S13. According to the results of OrthoMCL (Li et al., 2003), expansions and contractions of orthologous gene families were detected using CAFE (De Bie et al., 2006), and enrichment tests were performed using information from the homologs in the GO (The Gene Ontology Consortium, 2017) and KEGG (Kanehisa et al., 2014) databases.

## Data Availability

The datasets presented in this study can be found in online repositories. The names of the repository/repositories and accession number(s) can be found in the article/Supplementary Material.
