# Supplementary material for: Chromosome-Level Genome Assembly of the Asian Red-Tail Catfish (Hemibagrus wyckioides)
Source: Front Genet. 2021 Oct 12;12:747684. doi: 10.3389/fgene.2021.747684 (PMC8546334; doi:10.3389/fgene.2021.747684)
Supplement: Supplementary file 1 [file DataSheet1.docx]

**Supplementary File**

Chromosome-level genome assembly of the red-tail catfish (*Hemibagrus wyckioides*)

Feng Shao^1, *^, Huamei Pan^1^, Ping Li^1, 2^, Luyun Ni^1^, Yuan Xu^1^, Zuogang Peng^1, *^

^1^ Key Laboratory of Freshwater Fish Reproduction and Development (Ministry of Education), Southwest University School of Life Sciences, Chongqing 400715, China

^2^ College of Fisheries, Southwest University, Chongqing 400715, China

Table S1. Data usage details.

| Library | Library strategy | Design description | Size (Gb) |
| --- | --- | --- | --- |
| PromethION of *Hemibagrus wyckioides*: Blood | WGS | genome assemble | 74.9 |
| PacBio Sequel II of *Hemibagrus wyckioides*: Blood | WGS | genome assemble | 6.7 |
| NGS of *Hemibagrus wyckioides*: Blood | Hi-C | genome assemble | 82.7 |
| NGS of *Hemibagrus wyckioides*: Blood | WGS | survey and polishing | 41.0 |
| RNA-Seq (PromethION) of *Hemibagrus wyckioides*: Pooled | RNA-Seq | annotation | 17.3 |
| RNA-Seq of *Hemibagrus wyckioides*: Liver | RNA-Seq | annotation | 7.13 |
| RNA-Seq of *Hemibagrus wyckioides*: Heart | RNA-Seq | annotation | 10.6 |
| RNA-Seq of *Hemibagrus wyckioides*: Brain | RNA-Seq | annotation | 6.8 |
| RNA-Seq of *Hemibagrus wyckioides*: Gill | RNA-Seq | annotation | 7.6 |
| RNA-Seq of *Hemibagrus wyckioides*: Muscle | RNA-Seq | annotation | 8.2 |
| RNA-Seq of *Hemibagrus wyckioides*: Kidney | RNA-Seq | annotation | 9.0 |
| RNA-Seq of *Hemibagrus wyckioides*: Spleen | RNA-Seq | annotation | 7.9 |
| RNA-Seq of *Hemibagrus wyckioides*: Head kidney | RNA-Seq | annotation | 7.1 |
| RNA-Seq of *Hemibagrus wyckioides*: Fins | RNA-Seq | annotation | 7.4 |

Table S2. The statistics of *Hemibagrus wyckioides* genome assembly results based on long reads.

| Stat Type | Preliminary Assembly | | Polish Genome | |
| --- | --- | --- | --- | --- |
|  | Contig Length (bp) | Contig Number | Contig Length (bp) | Contig Number |
| N50 | 21,944,952 | 15 | 22,081,342 | 15 |
| Longest | 37,677,470 | 1 | 37,914,308 | 1 |
| Total | 784,971,716 | 176 | 789,788,867 | 176 |

Table S3. Assessment of *Hemibagrus wyckioides* genome completeness by BUSCO.

| Type | Number | Percent (%) |
| --- | --- | --- |
| Complete BUSCOs (C) | 3,491 | 95.91 |
| Complete and single-copy BUSCOs (S) | 3,455 | 94.92 |
| Complete and duplicated BUSCOs (D) | 36 | 0.99 |
| Fragmented BUSCOs (F) | 22 | 0.60 |
| Missing BUSCOs (M) | 127 | 3.49 |
| Total BUSCO groups searched | 3,640 | 100.00 |

Table S4. The results of RNA-seq reads aligned to the assembly.

| Sample name | Total reads | Total mapped | Multiple mapped | Uniquely mapped |
| --- | --- | --- | --- | --- |
| Liver | 47238678 | 43817335 (92.76%) | 2256863 (4.78%) | 41560472 (87.98%) |
| Kidney | 59022362 | 52955416 (89.72%) | 2043591 (3.46%) | 50911825 (86.26%) |
| Brain | 44846964 | 39515716 (88.11%) | 778073 (1.73%) | 38737643 (86.38%) |
| Muscle | 54947296 | 51971294 (94.58%) | 5805891 (10.57%) | 46165403 (84.02%) |
| Heart | 69946440 | 62765698 (89.73%) | 1826300 (2.61%) | 60939398 (87.12%) |
| Spleen | 51988142 | 48917695 (94.09%) | 1595486 (3.07%) | 47322209 (91.03%) |
| Gill | 49919006 | 45595429 (91.34%) | 1911004 (3.83%) | 43684425 (87.51%) |
| Fins | 48855778 | 45511263 (93.15%) | 1794683 (3.67%) | 43716580 (89.48%) |
| Head kidney | 46886258 | 42988419 (91.69%) | 1791902 (3.82%) | 41196517 (87.86%) |

Table S5. The statistics of chromosome length distribution based on Hi-C clustered of *Hemibagrus wyckioides* contigs.

| Group_ID | Length | Scaf Num |
| --- | --- | --- |
| LG01 | 35260361 | 7 |
| LG02 | 26507701 | 6 |
| LG03 | 35035625 | 3 |
| LG04 | 33136007 | 4 |
| LG05 | 30390217 | 4 |
| LG06 | 46190337 | 7 |
| LG07 | 34927831 | 8 |
| LG08 | 29551790 | 4 |
| LG09 | 31078239 | 7 |
| LG10 | 26000000 | 2 |
| LG11 | 34707240 | 4 |
| LG12 | 17012269 | 3 |
| LG13 | 27045622 | 2 |
| LG14 | 26010643 | 3 |
| LG15 | 24206193 | 6 |
| LG16 | 24083622 | 2 |
| LG17 | 26109670 | 4 |
| LG18 | 24815393 | 1 |
| LG19 | 24370555 | 5 |
| LG20 | 22209275 | 6 |
| LG21 | 21583638 | 6 |
| LG22 | 23013235 | 6 |
| LG23 | 20899033 | 6 |
| LG24 | 21142776 | 6 |
| LG25 | 21145816 | 3 |
| LG26 | 22786624 | 8 |
| LG27 | 20844914 | 4 |
| LG28 | 20838580 | 4 |
| LG29 | 20683148 | 5 |
| Total | 771586354 | 136 |

Table S6. The statistics of repeated sequences in *Hemibagrus wyckioides* genome.

| Class | Order | Super family | Number | Length (bp) | % of sequence |
| --- | --- | --- | --- | --- | --- |
| Class I TEs |  |  | 681,259 | 95,855,776 | 12.14 |
|  | LTR |  | 298,914 | 38,259,846 | 4.84 |
|  |  | Unknown | 122,826 | 15,892,715 | 2.01 |
|  |  | *ERV1* | 49,536 | 5,197,248 | 0.66 |
|  |  | *Gypsy* | 63,867 | 11,578,166 | 1.47 |
|  |  | *Copia* | 11,086 | 1,285,127 | 0.16 |
|  |  | *Ngaro* | 13,048 | 1,126,246 | 0.14 |
|  |  | *DIRS* | 9,155 | 1,583,447 | 0.20 |
|  |  | Other | 29,396 | 1,596,897 | 0.20 |
|  | SINE |  | 86,861 | 9,218,018 | 1.17 |
|  |  | Unknown | 31,328 | 3,080,975 | 0.39 |
|  |  | *MIR* | 22,797 | 3,239,507 | 0.41 |
|  |  | *tRNA-V* | 10,351 | 1,325,545 | 0.17 |
|  |  | Other | 22,385 | 1,571,991 | 0.20 |
|  | LINE |  | 295,484 | 48,377,912 | 6.13 |
|  |  | Unknown | 83,009 | 13,612,728 | 1.72 |
|  |  | *Rex-Babar* | 43,054 | 9,365,243 | 1.19 |
|  |  | *L1-Tx1* | 11,512 | 3,060,380 | 0.39 |
|  |  | *L2* | 77,452 | 10,701,017 | 1.35 |
|  |  | *RTE-BovB* | 15,306 | 4,214,213 | 0.53 |
|  |  | *L1* | 16,739 | 2,600,117 | 0.33 |
|  |  | *Penelope* | 20,698 | 2,198,440 | 0.28 |
|  |  | Other | 27,714 | 2,625,774 | 0.33 |
| Class II TEs |  |  | 1,270,899 | 150,776,164 | 19.09 |
|  | DNA |  | 1,196,841 | 140,419,570 | 17.78 |
|  |  | Unknown | 254,948 | 24,776,491 | 3.14 |
|  |  | *TcMar-Tigger* | 30,903 | 3,549,501 | 0.45 |
|  |  | *PIF-Harbinger* | 27,913 | 1,844,752 | 0.23 |
|  |  | *hAT-Tip100* | 26,943 | 2,168,115 | 0.27 |
|  |  | *CMC-EnSpm* | 101,148 | 7,432,103 | 0.94 |
|  |  | *TcMar-Tc1* | 236,518 | 52,591,902 | 6.66 |
|  |  | *hAT-Ac* | 60,768 | 5,099,899 | 0.65 |
|  |  | *Sola-3* | 36,503 | 7,955,619 | 1.01 |
|  |  | *Ginger* | 35,950 | 2,857,715 | 0.36 |
|  |  | *Maverick* | 45,786 | 2,670,193 | 0.34 |
|  |  | *hAT-Charlie* | 28,457 | 2,463,033 | 0.31 |
|  |  | *Zisupton* | 45,983 | 4,356,989 | 0.55 |
|  |  | *Sola-1* | 51,789 | 7,271,429 | 0.92 |
|  |  | *Kolobok-T2* | 19,370 | 1,214,200 | 0.15 |
|  |  | *Crypton-V* | 12,265 | 803,931 | 0.10 |
|  |  | *Kolobok-Hydra* | 13,246 | 971,769 | 0.12 |
|  |  | *Crypton-A* | 19,398 | 1,937,429 | 0.25 |
|  |  | *TcMar-Fot1* | 13,782 | 1,004,595 | 0.13 |
|  |  | *IS3EU* | 16,793 | 1,600,349 | 0.20 |
|  |  | Other | 118,378 | 7,849,556 | 0.99 |
|  | RC |  | 39,711 | 5,966,994 | 0.76 |
|  |  | Helitron | 39,711 | 5,966,994 | 0.76 |
|  | MITE |  | 34,347 | 4,389,600 | 0.56 |
|  |  | Unknown | 34,347 | 4,389,600 | 0.56 |
| Total TEs |  |  | 1,952,158 | 246,631,940 | 31.23 |
| Tandem Repeats |  |  | 315,023 | 23,614,957 | 2.99 |
|  | tandem_repeat |  | 123,473 | 20,870,444 | 2.64 |
|  | SSR |  | 191,550 | 2,744,513 | 0.35 |
| Unknown |  |  | 145,011 | 17,810,774 | 2.26 |
| Other |  |  | 48,431 | 8,704,195 | 1.10 |
| Simple repeats |  |  | 88,258 | 19,085,232 | 2.42 |
| Low complexity |  |  | 5,674 | 316,847,809 | 0.13 |
| Total Repeats |  |  | 2,554,555 | 316,847,809 | 40.12 |

Table S7. Summary of the results for BUSCO analysis according to *Hemibagrus wyckioides* gene prediction.

| Type | Number | Percent (%) |
| --- | --- | --- |
| Complete BUSCOs (C) | 3,443 | 94.59 |
| Complete and single-copy BUSCOs (S) | 3,388 | 93.08 |
| Complete and duplicated BUSCOs (D) | 55 | 1.51 |
| Fragmented BUSCOs (F) | 34 | 0.93 |
| Missing BUSCOs (M) | 163 | 4.48 |
| Total BUSCO groups searched | 3,640 | 100.00 |

Table S8. The statistics of functional annotated protein-coding genes in *Hemibagrus wyckioides* genome.

| Type | | Number | Percent (%) |
| --- | --- | --- | --- |
| Annotation | SwissProt | 19,841 | 87.04 |
|  | KEGG | 14,039 | 61.59 |
|  | KOG | 14,464 | 63.46 |
|  | GO | 14,503 | 63.63 |
|  | NR | 20,934 | 91.84 |
| Total | Annotated | 21,142 | 92.75 |
|  | Gene | 22,794 | - |

Table S9. Expansion gene families of female *Hemibagrus wyckioides* genome were enriched in 22 GO categories.

| ID | Description | GO_Class |
| --- | --- | --- |
| GO:0003810 | protein-glutamine gamma-glutamyltransferase activity | MF |
| GO:0018149 | peptide cross-linking | BP |
| GO:0042157 | lipoprotein metabolic process | BP |
| GO:0005525 | GTP binding | MF |
| GO:0006869 | lipid transport | BP |
| GO:0003723 | RNA binding | MF |
| GO:0016758 | transferase activity, transferring hexosyl groups | MF |
| GO:0008289 | lipid binding | MF |
| GO:0005506 | iron ion binding | MF |
| GO:0004950 | chemokine receptor activity | MF |
| GO:0016702 | oxidoreductase activity, acting on single donors with incorporation of molecular oxygen, incorporation of two atoms of oxygen | MF |
| GO:0006935 | chemotaxis | BP |
| GO:0016712 | oxidoreductase activity, acting on paired donors, with incorporation or reduction of molecular oxygen, reduced flavin or flavoprotein as one donor, and incorporation of one atom of oxygen | MF |
| GO:0008146 | sulfotransferase activity | MF |
| GO:0005576 | extracellular region | CC |
| GO:0003924 | GTPase activity | MF |
| GO:0008373 | sialyltransferase activity | MF |
| GO:0016705 | oxidoreductase activity, acting on paired donors, with incorporation or reduction of molecular oxygen | MF |
| GO:0008009 | chemokine activity | MF |
| GO:0020037 | heme binding | MF |
| GO:0006486 | protein glycosylation | BP |
| GO:0046983 | protein dimerization activity | MF |

MF: molecular function, BP: biological process, CC: cellular component.

Table S10. Expansion gene families of female *Hemibagrus wyckioides* were enriched in 33 KEGG pathways.

| ID | Description |
| --- | --- |
| map03013 | RNA transport |
| map03015 | mRNA surveillance pathway |
| map03018 | RNA degradation |
| map04630 | Jak-STAT signaling pathway |
| map04612 | Antigen processing and presentation |
| map00532 | Glycosaminoglycan biosynthesis - chondroitin sulfate / dermatan sulfate |
| map04113 | Meiosis - yeast |
| map00521 | Streptomycin biosynthesis |
| map00053 | Ascorbate and aldarate metabolism |
| map04111 | Cell cycle - yeast |
| map00040 | Pentose and glucuronate interconversions |
| map04145 | Phagosome |
| map00982 | Drug metabolism - cytochrome P450 |
| map00980 | Metabolism of xenobiotics by cytochrome P450 |
| map00860 | Porphyrin and chlorophyll metabolism |
| map04514 | Cell adhesion molecules (CAMs) |
| map00140 | Steroid hormone biosynthesis |
| map04218 | Cellular senescence |
| map00052 | Galactose metabolism |
| map04110 | Cell cycle |
| map00500 | Starch and sucrose metabolism |
| map00830 | Retinol metabolism |
| map04330 | Notch signaling pathway |
| map04061 | Viral protein interaction with cytokine and cytokine receptor |
| map04977 | Vitamin digestion and absorption |
| map04144 | Endocytosis |
| map00983 | Drug metabolism - other enzymes |
| map00051 | Fructose and mannose metabolism |
| map04973 | Carbohydrate digestion and absorption |
| map00520 | Amino sugar and nucleotide sugar metabolism |
| map04975 | Fat digestion and absorption |
| map00010 | Glycolysis / Gluconeogenesis |
| map04979 | Cholesterol metabolism |

Table S11. Contraction gene families of female *Hemibagrus wyckioides* genome were enriched in 19 GO categories.

| ID | Description | GO_Class |
| --- | --- | --- |
| GO:0005328 | neurotransmitter:sodium symporter activity | MF |
| GO:0006836 | neurotransmitter transport | BP |
| GO:0005886 | plasma membrane | CC |
| GO:0007156 | homophilic cell adhesion via plasma membrane adhesion molecules | BP |
| GO:0007154 | cell communication | BP |
| GO:0007155 | cell adhesion | BP |
| GO:0003774 | motor activity | MF |
| GO:0016459 | myosin complex | CC |
| GO:0005044 | scavenger receptor activity | MF |
| GO:0051015 | actin filament binding | MF |
| GO:0016567 | protein ubiquitination | BP |
| GO:0005887 | integral component of plasma membrane | CC |
| GO:0000786 | nucleosome | CC |
| GO:0008061 | chitin binding | MF |
| GO:0046982 | protein heterodimerization activity | MF |
| GO:0006352 | DNA-templated transcription, initiation | BP |
| GO:0004842 | ubiquitin-protein transferase activity | MF |
| GO:0004553 | hydrolase activity, hydrolyzing O-glycosyl compounds | MF |
| GO:0046872 | metal ion binding | MF |

MF: molecular function, BP: biological process, CC: cellular component.

Table S12. Contraction gene families of female *Hemibagrus wyckioides* were enriched in 9 KEGG pathways.

| ID | Description |
| --- | --- |
| map04928 | Parathyroid hormone synthesis, secretion and action |
| map04621 | NOD-like receptor signaling pathway |
| map04640 | Hematopoietic cell lineage |
| map04662 | B cell receptor signaling pathway |
| map04970 | Salivary secretion |
| map00520 | Amino sugar and nucleotide sugar metabolism |
| map04514 | Cell adhesion molecules (CAMs) |
| map04530 | Tight junction |
| map04721 | Synaptic vesicle cycle |

Table S13. Fossil calibration points of different species used in this study.

| Species 1 | Species 2 | Min  fossil time | Max  fossil time | Reference |
| --- | --- | --- | --- | --- |
| *L. oculatus* | *O. latipes* | 291 | 338 | Case & Schwimmer, 1988 |
| *O. latipes* | *D. rerio* | 205 | 255 | Hurley *et al*., 2007 |
| *D. rerio* | *E. electricus* | 126 | 179 | Zardoya & Doadrio, 1999 |
| *E. electricus* | *P. nattereri* | 116 | 176 | Near *et al*., 2012 |
| *B. yarrelli* | *G. maculatum* | 5.6 | 12.2 | Peng *et al*., 2006 |
| *I. punctatus* | *A. melas* | 40 | 58 | Wilson, 1967 |

Figure S1. Frequency distribution of the 17-mer graph analysis used to estimate the size of *Hemibagrus wyckioides* (black line) and *K-mer* curve (blue dotted line) fitting was used to estimate the heterozygosity of *Hemibagrus wyckioides*.


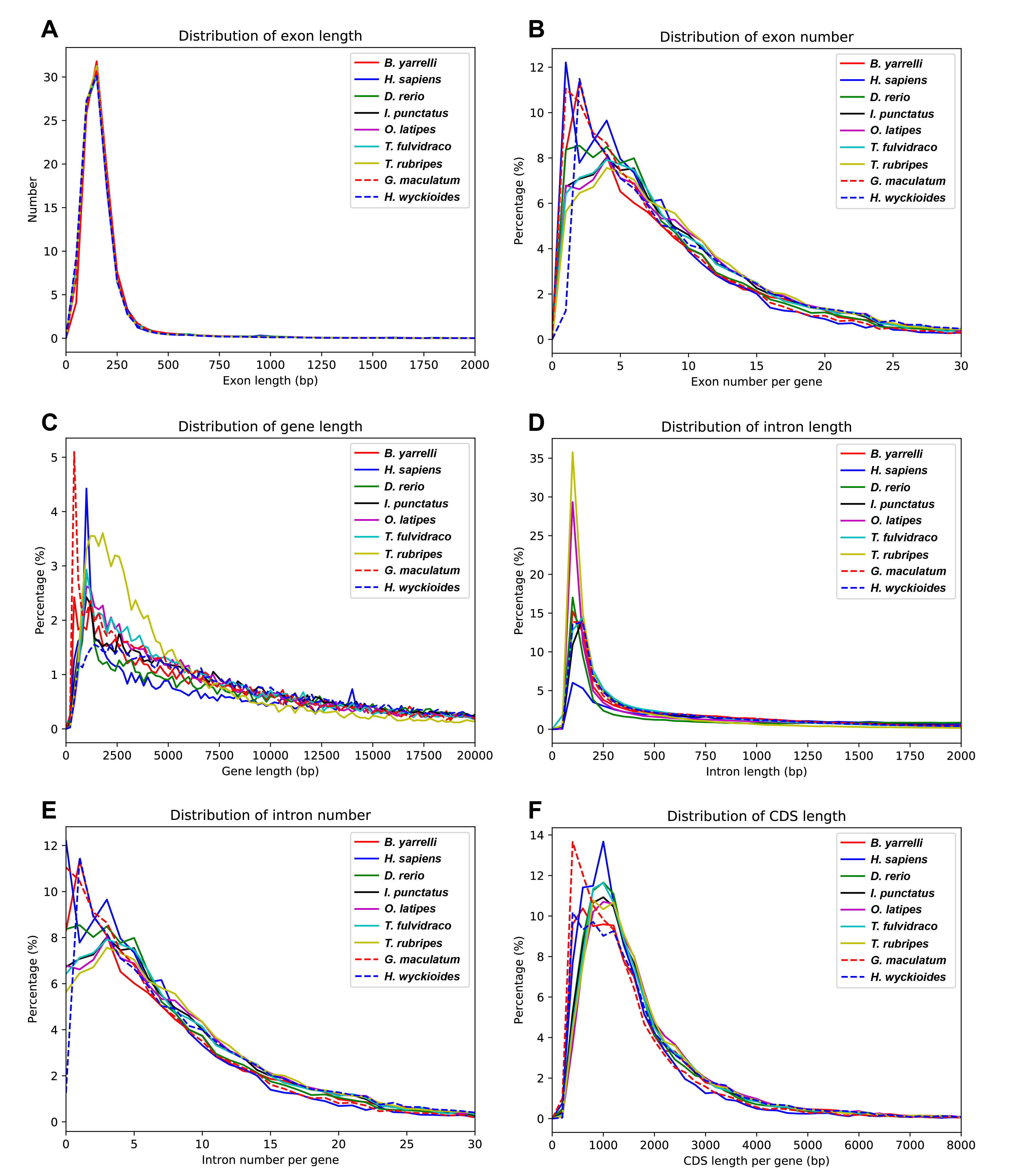


Figure S2. The comparisons of CDS length, exon length, exon-number, gene length, intro length, and intron number in genomes of female *Hemibagrus wyckioides* and other teleosts.

Figure S3. Divergence time of *Hemibagrus wyckioides* and other fish species.

**References:**

Case GR, Schwimmer DR. 1988. Late cretaceous fish from the blufftown formation (campanian) in western Georgia. Journal of Paleontology 62: 290-301.

Hurley IA, et al. 2007. A new time-scale for ray-finned fish evolution. Proceedings. Biological Sciences 274: 489-498.

Near TJ, et al. 2012. Resolution of ray-finned fish phylogeny and timing of diversification. Proceedings of the National Academy of Sciences USA 109: 13698-13703.

Peng ZG, Ho SYW, Zhang YG, He SP. 2006. Uplift of the Tibetan plateau: Evidence from divergence times of glyptosternoid catfishes. Molecular Phylogenetics & Evolution 39: 568–572.

Wilson RL. 1967. The Pleistocene vertebrates of Michigan. Papers of the Michigan Academy of Science, Arts, and Letters 52: 197-234.

Zardoya R, Doadrio I. 1999. Molecular Evidence on the Evolutionary and Biogeographical Patterns of European Cyprinids. Journal of Molecular Evolution 49: 227-237.
